# Use of Parsimony Analysis to Identify Areas of Endemism of Chinese Birds: Implications for Conservation and Biogeography

**DOI:** 10.3390/ijms11052097

**Published:** 2010-05-10

**Authors:** Xiao-Lei Huang, Ge-Xia Qiao, Fu-Min Lei

**Affiliations:** Key Laboratory of Zoological Systematics and Evolution, Institute of Zoology, Chinese Academy of Sciences, Beijing 100101, China; E-Mails: huangxl@ioz.ac.cn (X.-L.H); qiaogx@ioz.ac.cn (G.-X.Q)

**Keywords:** endemicity, areas of endemism, China, bird, parsimony analysis

## Abstract

Parsimony analysis of endemicity (PAE) was used to identify areas of endemism (AOEs) for Chinese birds at the subregional level. Four AOEs were identified based on a distribution database of 105 endemic species and using 18 avifaunal subregions as the operating geographical units (OGUs). The four AOEs are the Qinghai-Zangnan Subregion, the Southwest Mountainous Subregion, the Hainan Subregion and the Taiwan Subregion. Cladistic analysis of subregions generally supports the division of China’s avifauna into Palaearctic and Oriental realms. Two PAE area trees were produced from two different distribution datasets (year 1976 and 2007). The 1976 topology has four distinct subregional branches; however, the 2007 topology has three distinct branches. Moreover, three Palaearctic subregions in the 1976 tree clustered together with the Oriental subregions in the 2007 tree. Such topological differences may reflect changes in the distribution of bird species through *circa* three decades.

## 1. Introduction

China has a high degree of avian endemism, a large land area and a diverse range of habitats spanning both the Palaearctic and the Oriental realms. These features make China an important center of global biodiversity and a good place for biogeographical research. The patterns of distribution of endemic birds have previously been used to set biodiversity conservation priorities in China [1,2]. Distribution patterns of endemic bird species are also fundamental to determine China’s avifaunal regionalization [3,4], and to interpret subregional avian endemism [5]. BirdLife International has set up global endemic bird areas (EBAs) based on the distribution of species with restricted ranges [6], and areas of high endemism have been targeted for protection [6,7]. Sometimes, endemic birds are effective surrogates for identifying conservation priorities [8] because of similar distribution patterns with other vertebrate taxa [9]. Although some previous studies have analyzed distribution patterns in China of endemic birds from different aspects, there has been no report about their areas of endemism (AOEs). Identification of AOEs is important, not only for understanding evolution of Chinese avian fauna, but also for identifying hotspots of avian biodiversity conservation, and may serve as a starting point for developing conservation strategies for other vertebrate taxa.

Parsimony analysis of endemicity (PAE) is a biogeographical method first proposed by Rosen [10], which uses a parsimony algorithm to obtain area cladograms based on the geographical distributions of specified taxa [11]. PAE can be used to infer relationships among different biogeographical units (e.g., localities, quadrats, continents, islands) [12]. Although there is ongoing debate about the value of PAE, PAE has also proven to be a useful and important tool for identifying AOEs [12–15]. The most common procedure of PAE, as proposed by Morrone [13], used quadrats of predetermined size; and different authors have used different operating geographical units (OGUs) [16–21]. However, smaller quadrats may decrease the absolute number of steps in the area cladogram while larger quadrats may increase the number of synapomorphies. The use of ‘somewhat more natural’ OGUs, such as biogeographical provinces, subregions and ecoregions, instead of artificial quadrats, is thought to increase the absolute and relative numbers of synapomorphies [12,22]. Furthermore, such OGUs are useful to understand biogeographical evolution at a large scale. Therefore, we used avian subregions [3–5] as the most appropriate OGU in this PAE analysis.

While historical change in patterns of avian distribution has been documented [23,24], evidence of change over a more recent timescale is likely to be related to current climate change or human activities [25–29]. A previous paper tried to link the distribution changes of bird species in China with global warming [30], however, very limited data (only 12 new distribution records) was used. PAE can produce an area tree by using species distributions as characters in parsimony analysis. Thus, it may give us a chance to investigate distribution shift by comparing area topologies from different datasets (change of character assignment in parsimony analysis will be reflected in the area topology). The data accumulation over more than half a century in our lab [5,31] is a good basis to investigate the relationship between bird distribution shift and global change (for details about the databases see the Experimental Section).

To better understand the evolution of avian fauna in China and to improve our knowledge in avian conservation, the present study aims to identify the areas of endemism for birds in China, and tries to search for some signs of distribution changes of Chinese endemic birds through *circa* three decades.

## 2. Results and Discussion

### 2.1. Results

#### 2.1.1. Subregional Avian AOEs

**Figure 1.**
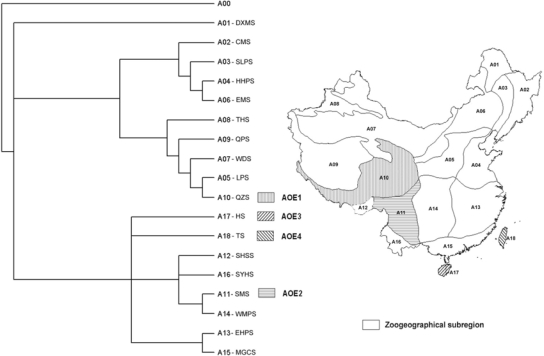
A tree of Chinese avian subregions based on a 2007 dataset comprised of the distributions of 105 endemic bird species in 18 subregions. The avian subregional codes are: A00 = the hypothetical outgroup, A01 = Da Xinganling Mountain Subregion (DXMS), A02 = Changbai Mt. Subregion (CMS), A03 = Song-Liao Plain Subregion (SLPS), A04 = Huang-Huai Plain Subregion (HHPS), A05 = Loess Plateau Subregion (LPS), A06 = East Meadow Subregion (EMS), A07 = West Desert Subregion (WDS), A08 = Tianshan Hilly Subregion (THS), A09 = Qiangtang Plateau Subregion (QPS), A10 = Qinghai-Zangnan Subregion (QZS), A11 = Southwest Mountainous Subregion (SMS), A12 = Southeast Himalayan Slope (SHSS) Subregion, A13 = Eastern Hillock-Plain Subregion (EHPS), A14 = Western Mountainous Plateau Subregion (WMPS), A15 = Min-Guang Coastal Subregion (MGCS), A16 = Southern Yunnan Hilly Subregion (SYHS), A17 = Hainan Subregion (HS), A18 = Taiwan Subregion (TS).

A tree derived from the 2007 data, comprised of 105 endemic bird species in 18 subregions, indicates that the 18 subregions form three main branches (Figure 1). The first branch is comprised of only the DXMS (avian subregional code A01; for the abbreviations for the subregions, refer to the caption of Figure 1). The second branch includes nine subregions, *i.e.*, A02–A10, all of which belong to the Palaearctic realm. The third branch includes eight subregions (A11–A18), all of which belong to the Oriental realm. With the exception of the DXMS, this PAE-derived topology reflects the accepted division of China’s avifauna into Palaearctic and Oriental realms.

Four AOEs were identified at the subregional level (Figure 1). Of these subregions, one is in the Palaearctic realm and the other three are in the Oriental realm. AOE1 is the Qinghai-Zangnan Subregion (clade A10) on the southeastern edge of the Tibetan Plateau, and was delimited by the species *Alectoris magna*, *Babax koslowi*, *Garrulax sukatschewi*, *Kozlowia roborowskii* and *Paradoxornis przewalskii*. AOE2 is the Southwest Mountainous Subregion (clade A11) delimited by *Arborophila rufipectus*, *Garrulax bieti*, *Liocichla omeiensis*, *Moupinia poecilotis*, *Paradoxornis zappeyi* and *Phylloscopus emeienses*. AOE3 is Hainan Subregion (clade A17) delimited by *Arborophila ardens* and *Phylloscopus hainanus*. AOE4 is the Taiwan Subregion (clade A18) delimited by *Actinodura morrisoniana*, *Arborophila crudigularis*, *Bradypterus alishannensis*, *Garrulax morrisonianus*, *Heterophasia auricularis*, *Liocichla steerii*, *Lophura swinhoii*, *Myiophoneus insularis*, *Parus holsti*, *Pycnonotus taivanus*, *Regulus goodfellowi*, *Syrmaticus mikado*, *Tarsiger johnstoniae*, *Urocissa caerulea* and *Yuhina brunneiceps*. The greater number of species delimiting this AOE reflects the fact that it has the highest endemicity of all four AOEs.

#### 2.1.2. The 1976 and the 2007 Subregional Topologies

**Figure 2.**
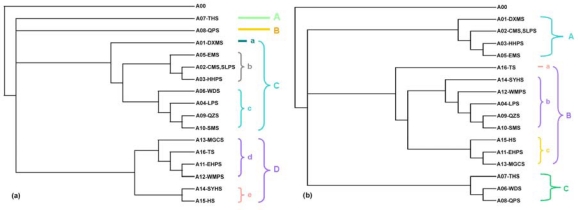
**(a)** Dendrogram of Chinese avian subregions based on a 1976 dataset comprised of the distributions of 99 endemic bird species in 16 subregions. The avian subregional codes are: A01 = DXMS, A02 = CMS and SLPS, A03 = HHPS, A04 = LPS, A05 = EMS, A06 = WDS, A07 = THS, A08 = QPS, A09 = QZS, A10 = SMS and SHSS, A11 = EHPS, A12 = WMPS, A13 = MGCS, A14 = SYHS, A15 = HS, A16 = TS. **(b)** Dendrogram of subregions based on a 2007 dataset comprised of the distributions of the same 99 species in 16 subregions as in (a).

The 1976 subregional topology from the PAE analysis has four distinctive branches (A, B, C and D) (Figure 2a). With one exception, most subregions from the Oriental realm are clustered in branch D and those from the Palaearctic realm in branches A, B, and C. Branch A and B have only one subregion each. Branch C and D can be divided into minor subregional branches as Figure 2a shows. Branch A has only one endemic species, *Podoces hendersoni*, branch B has five endemic species. In branch C, group *a* has one subregion and only one species, *Garrulax davidi*, which is co-distributed in all but one of the other subregions of branch C. Four and five subregions belong to group *b* and *c*, respectively. Group *d*, which has four subregions, is clustered with group *e*, with two subregions, to form branch D.

The 2007 subregional topology has three distinct branches (Figure 2b). All subregions from the Oriental realm, together with three from the Palaearctic realm, are clustered in branch B. The other subregions from the Palaearctic realm form the other two branches, A and C. Branch B contains three groups, among which branch *a* has only one subregion (A16) with very typical island habitat.

#### 2.1.3. Topological Differences between the Two Trees

**Figure 3.**
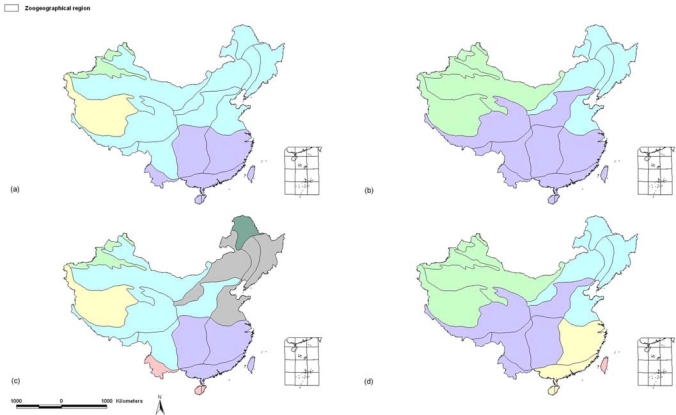
Sketch of subregional topologies based on Figure 2. **(a)** sketch based on the 1976 dataset as in Figure 2a at the four terminal branches scale; **(b)** based on the 2007 dataset as in Figure 2b at the three terminal branches scale; **(c)** based on Figure 2a at the seven group scale; **(d)** based on Figure 2b at the five group scale.

Comparison of the topologies derived from the 1976 and 2007 data indicates that: 1) on a large scale (Figure 3a,b) some central and southern clades have mixed to form a larger southern region, however, in contrast, northeastern clades have contracted within a smaller region; 2) on a fine scale (Figure 3c,d) southeastern clades have combined to form a larger region while some clades in northeastern China have mixed to form a larger branch; 3) three Palaearctic subregions in the 1976 tree clustered together with the Oriental subregions in the 2007 tree.

### 2.2. Discussion

#### 2.2.1. Areas of Endemism at Subregional Scale

The general topology based on distributions of the 105 species revealed three main branches: the DXMS, the Palaearctic subregions, and the Oriental subregions. Among them, the DXMS has only the one species, *Garrulax davidi*. It is very probable that such little information caused this branch to separate from the other Palaearctic subregions. Except this branch, the subregional topology generally reflects the accepted division between the Palaearctic and Oriental realms in China [3,4].

By using the PAE method, four avian AOEs at subregional level, *i.e.,* the Qinghai-Zangnan Subregion (AOE1), the Southwest Mountainous Subregion (AOE2), the Hainan Subregion (AOE3), and the Taiwan Subregion (AOE4) were identified (Figure 1). The Qinghai-Zangnan Subregion extends from the north Qilianshan mountains of northeastern Qinghai Province southward to Changdu and encompasses central and eastern regions of the Himalayan Mountains, including the Yaluzangbu valley of Tibetan Autonomous Region. Typical habitats in this area are montane forest (coniferous and broad-leaved), meadow and meadow-steppe. The Southwest Mountainous Subregion encompasses the western areas of Yunnan and Sichuan provinces and mostly covers the Hengduan mountain areas. Its typical habitats are mountain meadow and mountain forest with obvious altitudinal variation. The Hainan Subregion is confined to the Hainan Island. The typical habitat is tropical monsoon rain forest. The Taiwan Subregion is confined to the Taiwan Island and its offshore islands. Typical habitats are tropical monsoon rain forest and subtropical ever-green broad-leaved forest.

Although the Qinghai-Zangnan Subregion and the Southwest Mountainous Subregion are geographically contiguous on the southeast Tibetan Plateau, they are in two distinct separated branches in the tree, which is consistent with the division between the Palaearctic and Oriental realms [3,4]. The Qinghai-Zangnan Subregion is a mountainous area with habitats ranging from high mountain forests to meadow and steppe [4,32]. Apart from the local endemics, which defined this AOE, *G. sukatschewi*, *K. roborowskii*, *B. koslowi*, *Urocynchramus pylzowi* and *Emberiza koslowi* are also mostly restricted to this subregion. The Southwest Mountainous Subregion is also a mountainous area in the subtropical zone with high mountain ranges and deep valleys such as the Yaluzangbu Valley [3]. Besides the endemics restricted to this AOE, *A. rufipectus*, *M. poecilotis*, *G. bieti*, *L. omeiensis*, *P. zappeyi*, *P. emeienses*, *Arborophila rufipectus*, *Lophophorus sclateri*, *Crossoptilon harmani* and *Moupinia poecilotis* are also mostly confined to this subregion. AOEs are important areas for understanding fauna evolution, which has been determined by historical and ecological factors. For these two adjacent AOEs, they have related but discrepant geological history. As part of the uplift of the Tibetan Plateau and the Himalayas region, which began in the Late Cenozoic [32], the Qinghai-Zangnan region has been formed due to the recent and violent uplift (less than 2.5 million years ago). Harsh environmental pressures during this uplift mainly determined the fauna evolution in this region [15,33]. However, the Southwest Mountainous region has much longer geological history and was more stable during this geological event [4,32]. The differences between geological history, topography and ecological factors may determine this different endemic avian fauna of the two AOEs, with the inferred hypothesis that historical and ecological vicariant events may act as important factors in shaping this deep divergence between the two AOEs.

The other two AOEs, the Hainan and Taiwan subregions, are islands with typical mountainous habitats in the southern subtropical, northern and middle tropical zones. Mountains in the Hainan subregion are relatively low, the highest peak, Wuzhishan, is less than 2,000 m, and the average temperature is >25 °C. The Taiwan Subregion is more geographically isolated by the wider Taiwan Strait. Its western coastal areas are plains, but eastern and central parts are mountainous, with the highest mountain in southeastern China, Mt Yushan (3,997 m) [3]. Fifteen endemic species restricted to this region defined this AOE. For these two AOEs, especially for the Taiwan subregion, species diversification after geological isolation from the mainland may contribute to its current endemic avian fauna. Grey-cheeked Fulvetta (*Alcippe morrisonia*) populations [34,35] and Hwamei (*Garrulax canorus*) [36], for example, are well confirmed due to this isolation between island and mainland through a phylogeographical approach.

All the four AOEs have large part of mountainous habitats, which suggests that mountainous environment may act as “historical and ecological barriers” preventing population gene flow, promoting speciation and maintaining a high endemism [5,37–38].

#### 2.2.2. The Importance of AOEs for Avian Biodiversity Conservation

Lei *et al.* [5] documented the subregional avian endemism of China based on the number of endemic species, monotypic species and EOSR (species distributed only in one specific subregion). This approach may not reflect possible historical relationships and congruence in species distribution among different avian subregions. However, some results in the present paper are nearly congruent with those of Lei *et al.* [5], in that the Taiwan subregion had the highest EOSR species richness. This AOE, with an extremely high degree of regional endemism, reflects its particular geological, evolutionary and ecological isolation.

Of the four AOEs identified in this paper, three are spatially congruent with the avian biodiversity hotspots in China previously mapped by Lei *et al.* [1,2] (the Hengduanshan Mountains; the Western Qinling Mountains, north Sichuan and south Gansu provinces; Taiwan Island). AOE 2 nearly covers the Hengduan mountain areas, which is one of 25 global biodiversity hotspots [7] and China’s avian ‘evolutionary powerhouse’ [38]. To promote avian biodiversity conservation, BirdLife International has used other criteria to set up global endemic bird areas (EBAs). EBAs are defined as areas containing two or more species with restricted distributions and can overlap an adjoining EBA by no more than 50,000 km^2^ [6]. Most of the 13 EBAs and three secondary areas of avian biodiversity in China listed by BirdLife International [39] are located within the four AOEs described here.

AOEs are important for avian biodiversity conservation based on two main reasons: 1) according to its theoretical basis, AOEs are historically key areas for evolution of the avian fauna (namely, formation of avian biodiversity); 2) AOEs have a higher degree of avian endemism, especially endemic bird species with very restricted distributions. Our results suggest that the Southwest Mountainous Subregion and Taiwan Subregion have the highest conservation priority in terms of biodiversity, biogeography and evolution.

#### 2.2.3. Can We Infer Species Distribution Changes by PAE?

Some topology differences are indicated by comparison of the 1976 and 2007 topologies (Figures 2 and 3). According to the principle of the PAE analysis (parsimony analysis of the species distributions), topology changes indicate that species composition of the related subregions have changed over *ca*. three decades. One difference is that more subregions have become clustered to form larger branches. This implies that species dispersal has occurred between some subregional faunas. The group *a* (Taiwan island) (Figure 2b) has become more isolated from branch *d* (Figure 2a), which may suggest a recent decrease in migration between populations on Taiwan island and the Chinese mainland. The most distinct difference is that three Palaearctic subregions in the 1976 tree cluster together with the Oriental subregions in the 2007 tree, which indicates change of species composition of the three subregions, more specifically, oriental species may have dispersed to the Palaearctic subregions. Of the three subregions, the Huang-Huai Plain Subregion (A04) is located in eastern China, which has usually been considered as a dispersal corridor for animal species between southern and northern China. The other two subregions, Qiangtang Plateau Subregion (A09) and Qinghai-Zangnan Subregion (A10) are located on the Tibetan Plateau, which is the highest area of China and most sensitive to climate change. When we refer to the data about climate change (e.g., global climate warming) in China, it was found that more and more data suggest that during the past *ca.* half century, most regions of China have been experiencing climate warming, especially in regard to the winter mean temperature in eastern China [40,41], and the northern boundary of the subtropical zone moved 2–3 degrees of latitude northward in central and eastern China [42]. Moreover, a distinct tendency of increasing temperatures, precipitation and relative humidity over the Tibetan Plateau was reported, and data showed the Tibetan Plateau is more sensitive to the global change [43]. Based on these evidences, our data may suggest that south to north switches in trend across the boundary between the Palaearctic and Oriental realms in eastern China are probably happening, the bird species *Garrulax canorus, G. elloitii, Carpodacus trifasciatus,* and *Urocynchramus pylzowi* are good examples. Another type of distribution shift may happen on the Tibetan Plateau region, that is to say, some avian species are moving upward from Oriental subregions with relatively low elevation to the higher elevation of the plateau, such as *Parus davidi, Yuhina diademata, Alcippe variegaticeps* and *Paradoxornis przewalskii*.

When species are forced to move to different areas, so-called “optimal” habitats for many species may no longer exist, at least in the short term [44], and this may lead to local extinction of some species [45]. The subregional distribution shifts revealed in this paper encourages us to further investigate whether the distribution changes of bird species can exactly link with global warming, or other factors such as human activities or sampling bias. Furthermore, difference between mobility of species may also influence distribution patterns. Detailed investigations from more species and based on more detailed geographical scales are needed in the future.

## 3. Experimental Section

### 3.1. Species and Distributions

We used distributions of Chinese endemic bird species as dataset in the present paper for two reasons: 1) using endemic species can ensure that the distributional ranges of all species were included in the study area, and thus avoided problems related to partial distributional ranges in PAE analysis [46]; 2) the endemic species in China have been better studied than most other species and therefore have the most accurately mapped distributions [47].

The updated distribution database of endemic birds was from Lei *et al.* [5] (hereafter “2007 data”), which was compiled from distribution records before year 2005. China currently has 19 recognized avian subregions belonging to seven regions and two realms [3,4]. However, because there are almost no distribution records from the South-Sea Island Subregion, we only used the other 18 subregions in the PAE analysis.

Cheng’s Complete Checklist of the Avifauna of China [31] (hereafter “1976 data”) contains almost all past information on the distributions of Chinese birds before year 1974, which therefore provides a historical baseline of avian distribution in China. This monograph only provides data on 99 species in 16 subregions. To allow comparison between the two time periods, distributions of the same 99 species and in 16 subregions were used to derive trees from the 1976 and 2007 dataset.

### 3.2. Parsimony Analysis of Endemicity

Areas of endemism (AOEs) are usually defined as areas delimited by the congruent distribution of two or more species of restricted range [13,16,48]. Sympatry of different species may indicate a shared biological history [10,11]. The procedure of PAE analysis modified by Morrone [13] was used in the present study. For identifying AOEs, the 2007 data was used and a matrix of 18 OGUs (subregions) × 105 taxa (bird species) was constructed. To obtain comparable area trees, two matrices with the same 16 OGUs and 99 taxa were constructed from the 1976 and 2007 data. All matrices coded taxa as presence (1) or absence (0). Each matrix also included a hypothetical outgroup OGU with all 0s to root the trees [12,13]. PAE was implemented using PAUP 4.0 [49]. The strict consensus trees of the equally parsimonious trees in each analysis were obtained. AOEs, defined by two or more species being restricted to a group of OGUs, were delimited and mapped. Furthermore, two area trees of the two time periods were compared to see if we could find distribution shifts of bird species through time (*ca.* 30 years). For more details about PAE implementation see Huang *et al.* [15].

## 4. Conclusions

PAE was firstly used to identify areas of endemism of Chinese birds at the subregional level. Four AOEs, *i.e.,* Qinghai-Zangnan Subregion, the Southwest Mountainous Subregion, the Hainan Subregion and the Taiwan Subregion, were identified. The congruent environments of these AOEs indicate that mountainous environments may act as “historical and ecological barriers” in preventing population gene flow, promoting speciation and maintaining a high endemism, but the differences between geological history, topography and ecological factors may determine the vicariance between the former two AOEs. The cladistic analysis of subregions generally supports the division of China’s avifauna into Palaearctic and Oriental realms. PAE based topological differences produced from two different distribution datasets may reflect the distribution changes of bird species through *ca.* three decades. PAE are also found to be useful in avian conservation priority in terms of biodiversity, biogeography and evolution through identifying AOEs.
